# The immunomodulatory potential of the arylmethylaminosteroid sc1o

**DOI:** 10.1007/s00109-020-02024-4

**Published:** 2020-12-17

**Authors:** Leonard Blum, Thomas Ulshöfer, Marina Henke, Reimar Krieg, Isabell Berneburg, Gerd Geisslinger, Katja Becker, Michael J. Parnham, Susanne Schiffmann

**Affiliations:** 1 Fraunhofer Institute for Molecular Biology and Applied Ecology IME, Branch for Translational Medicine and Pharmacology (TMP), Theodor-Stern-Kai 7, 60596 Frankfurt am Main, Germany; 2 pharmazentrum frankfurt/ZAFES, Department of Clinical Pharmacology, Goethe-University Hospital Frankfurt, Theodor-Stern-Kai 7, 60590 Frankfurt/Main, Germany; 3 Department of Anatomy II, University Hospital Jena, Teichgraben 7, 07743 Jena, Germany; 4 Biochemistry and Molecular Biology, Interdisciplinary Research Center, Justus-Liebig-University, Heinrich-Buff-Ring 26-32, 35392 Giessen, Germany

**Keywords:** Steroid compound 1o, Macrophages, Dendritic cells, Immune modulation, Immune metabolism, *Plasmodium falciparum*

## Abstract

**Abstract:**

Developing resistance mechanisms of pathogens against established and frequently used drugs are a growing global health problem. Besides the development of novel drug candidates per se, new approaches to counteract resistance mechanisms are needed. Drug candidates that not only target the pathogens directly but also modify the host immune system might boost anti-parasitic defence and facilitate clearance of pathogens. In this study, we investigated whether the novel anti-parasitic steroid compound 1o (sc1o), effective against the parasites *Plasmodium falciparum* and *Schistosoma mansoni*, might exhibit immunomodulatory properties. Our results reveal that 50 μM sc1o amplified the inflammatory potential of M1 macrophages and shifted M2 macrophages in a pro-inflammatory direction. Since M1 macrophages used predominantly glycolysis as an energy source, it is noteworthy that sc1o increased glycolysis and decreased oxidative phosphorylation in M2 macrophages. The effect of sc1o on the differentiation and activation of dendritic cells was ambiguous, since both pro- and anti-inflammatory markers were regulated. In conclusion, sc1o has several immunomodulatory effects that could possibly assist the immune system by counteracting the anti-inflammatory immune escape strategy of the parasite *P. falciparum* or by increasing pro-inflammatory mechanisms against pathogens, albeit at a higher concentration than that required for the anti-parasitic effect.

**Key messages:**

• The anti-parasitic steroid compound 1o (sc1o) can modulate human immune cells.

• Sc1o amplified the potential of M1 macrophages.

• Sc1o shifts M2 macrophages to a M1 phenotype.

• Dendritic cell differentiation and activation was ambiguously modulated.

• Administration of sc1o could possibly assist the anti-parasitic defence.

**Supplementary Information:**

The online version contains supplementary material available at 10.1007/s00109-020-02024-4.

## Introduction

Increasingly resistant pathogens are causing a global health problem with a variety of infectious diseases. The most important treatments for parasite-mediated diseases such as malaria (*Plasmodium)* and schistosomiasis (*Schistosoma)* are artemisinin combination therapies for malaria and praziquantel for schistosomiasis. The WHO reported 228 million cases of malaria worldwide in 2018 [1], while schistosomiasis affects approximately 200–250 million people, mostly in developing countries [2–4]. The frequent use of the available drugs increases the risk of resistance mechanisms. Reports relating to artemisinin-resistant parasites stress the urgent need for new therapeutic approaches [5, 6]. Besides resistance mechanisms against the drugs, *Plasmodium* further accentuates the challenge as it reduces the defensive pro-inflammatory conditions of the host by promoting the M2-phenotype of monocytes, probably through haemozoin-induced CD206 expression [7, 8].

In addition to the direct impact of new drug candidates on pathogens, maintenance and promotion of immune responses are also important to overcome emerging pathogen resistance. Modulating the immune system is a promising approach to boost host defence mechanisms and increases the clearance of pathogens while minimizing tissue damage from inflammation. It has been shown that several antibiotics are able to modulate inflammatory processes and thereby promote pathogen defence even in the face of mechanisms to resist the direct antimicrobial impact of the drugs [9]. Therefore, immunomodulation can expand the efficacy profile and may unfold new therapeutic indications as a pro- or anti-inflammatory modulator.

Inflammation accompanies the majority of infections, regardless of whether they are caused by bacteria or parasites, and is characterized by the accumulation of various immune cells such as neutrophils, dendritic cells, monocytes, and macrophages at the site of infection [10]. This can result in the release of a variety of lipid mediators, cytokines, chemokines, growth factors, and enzymes that mediate pathogen killing but can also lead to bystander tissue injuries. In order to prevent an exaggerated immune response and protect the host tissue, a well-timed resolution of the inflammatory process is mandatory. Macrophages are essential for the local initiation of inflammation since they release several cytokines such as interleukin (IL)-1β, interferon (IFN)-γ, IL-23, and tumour necrosis factor (TNF)-α. Furthermore, they recruit additional immune cells by secreting chemokines such as CC-chemokine ligand (CCL)2, C-X-C motif chemokine (CXCL)10, and CXCL8 [11]. Besides activating the inflammatory process, macrophages recognize and ingest pathogens and activate T cells via HLA-DR. During the resolution of inflammation, macrophages release growth factors, cytokines (e.g. IL-10 and IL-4), and chemokines (e.g. CCL18 and CCL17) to recruit anti-inflammatory T_H_2 and T_reg_ cells to support the tissue healing process [11–14]. The immune response is regulated not only by macrophages but also by dendritic cells. The expression of specific surface markers such as CD40, CD80, CD86, and HLA-DR is essential for the presentation of antigens and the regulation of the antigen-specific T cell response by dendritic cells. Since macrophages and dendritic cells are important players in the fight against *Plasmodium falciparum* [15], we focused on these cell types. In malaria, excessive production of inflammatory cytokines, including TNF-α, IL-6, IL-12, and IFN-γ, at the early stages of infection is a key contributor to pathogenesis [16]. Mouse studies revealed that specifically dendritic cells are responsible for the cytokine release [17]. M1 macrophages form the first line in host defence against parasites. Since macrophages are highly adaptive to their local microenvironment and have been shown to switch their programming from one functional phenotype to another in response to local signals and M2 macrophages are thought to play an important role in maintaining the balance between inflammation and the restoration of tissue homeostasis after infection and local injury, we focused on both phenotypes.

Approved antibiotics with immunomodulatory effects include azithromycin and quinolones. Quinolones such as moxifloxacin interact with the immune response in a biphasic mode [18]. They initially induce the release of pro-inflammatory cytokines, phagocytosis, and the oxidative burst [19], subsequently leading to a halt in cytokine production. The steroid compound 1o (sc1o), a new lead compound with a promising activity profile against *Plasmodium falciparum* and *Schistosoma mansoni* parasites [20] and a good safety profile [21], consists of a steroid and a 2-hydroxyarylmethylamino moiety [20]. The 2-hydroxymethylamino residue is accessible to oxidative transformations that lead to quinone methide intermediates, which have some (formal) structural similarities with quinolones. Therefore, we speculated that sc1o might also interact with the immune system, and in this study, we have investigated some potential immunomodulatory effects of sc1o using standard stimuli. With this aim in mind, we tested the impact of sc1o on differentiation and polarization/activation of human monocyte-derived macrophages (MdM) and dendritic cells (MdDC) in vitro.

## Results

### Sc1o did not reduce cell viability in myeloid cells

Beside the specificity and potency of an antimicrobial drug, another prerequisite is that it is not cytotoxic to myeloid cells. We thus investigated whether sc1o influences cell viability of primary myeloid cells such as MdMs and MdDCs.

**Fig. 1:**
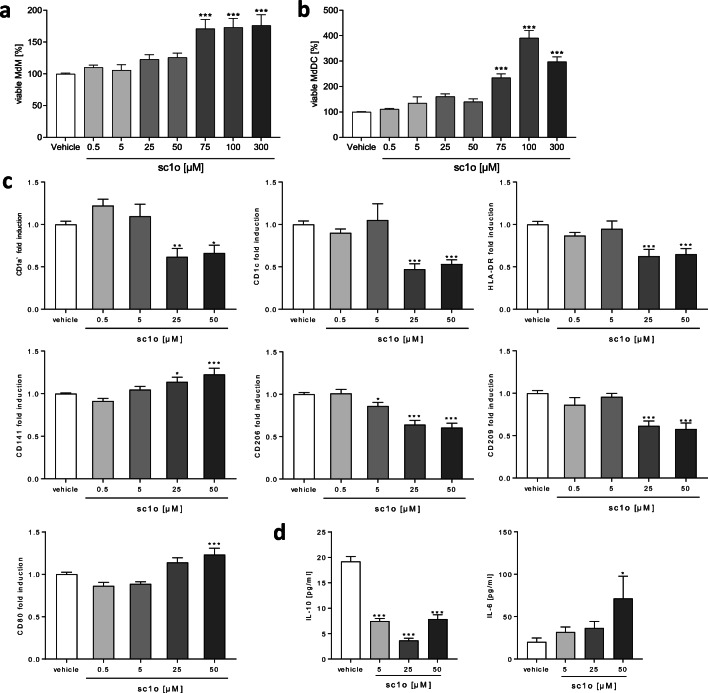
Sc1o influences myeloid cell viability and dendritic cell differentiation. The percentage of viable human monocyte-derived macrophages (MdM) (**a**) and monocyte-derived dendritic cells (MdDC) (**b**) in the presence or absence of different concentrations of sc1o (0.5–300 μM) or vehicle (DMSO) were determined with an Orangu™ assay in triplicate. Therefore, human CD14^+^ cells were isolated from buffy coats and differentiated to MdMs (7 days, 10 ng/ml GM-CSF) or MdDCs (5 days, 10 ng/ml GM-CSF and IL-4). After differentiation, cells were treated with different sc1o concentrations for 48 h. Viability was measured after 120 min incubation with the Orangu reagent. The percentage values were calculated with vehicle-treated cells as reference (*n* (**a**, **b**) = 3 blood donors in 1 experiment). The influence of sc1o on dendritic cells during differentiation (**c**/**d**). Human monocytes were isolated and differentiated to MdDCs for 5 days with GM-CSF (10 ng/ml) and IL-4 (10 ng/ml) in the presence or absence of different concentrations of sc1o (0.5–50 μM) or a vehicle (DMSO). (**c**) Surface marker expression was measured with a MACSQuant® Analyser 10 in triplicate. Fold induction of the geometric mean of the fluorescence intensity was calculated by referring treated cells to vehicle controls (*n* = 6–11 different blood donors in 4 separate experiments). (**d**) Released concentrations of IL-6 and IL-10 in the supernatant were measured with a cytometric bead array in triplicate (*n* = 4 different blood donors in 3 separate experiments). For statistical analysis, a one-way ANOVA with Dunnett’s multiple comparisons test (**a**–**d**) was used. Results are presented as means ± standard errors. * *p* < 0.05. ** *p* < 0.01. ***p < 0.001

Unexpectedly, sc1o increased the metabolic activity of MdMs and MdDCs tested by the Orangu™ assay, which measures the dehydrogenase activity in living cells using a tetrazolium substrate (Fig. 1a and b). Concentrations above 75 μM significantly increased metabolic activity in both cell types. The strongest effects could be detected in MdDCs with an increase of up to approximately 400% at 100 μM sc1o. Since higher concentrations are physiologically less relevant, a maximum concentration of 50 μM sc1o was used in the following experiments to test the immunomodulatory potential.

### Sc1o influenced the differentiation of dendritic cells

To test whether sc1o influences the process of dendritic cell differentiation, isolated monocytes were differentiated in the presence of different concentrations of sc1o (0.5–50 μM) or vehicle (DMSO). As a readout, surface markers that are regulated during differentiation were determined. Furthermore, released cytokines and chemokines were detected. Our results showed that the incubation with sc1o results in pro- and anti-inflammatory changes in MdDCs (Fig. 1c–d). The antigen-presenting surface markers (CD1a, CD1c, HLA-DR) and receptors essential for the phagocytosis of pathogens (CD206 and CD209) were downregulated by 25 und 50 μM sc1o. However, 50 μM sc1o significantly upregulated CD141 and the receptor CD80, essential for the activation of T cells, during the differentiation process (Fig. 1c). Sc1o did not regulate CD54, CD40, CD83, CD197, and CD86 (Supplemental Fig. 1a) and decreased the release of IL-10 (Fig. 1d). IL-6 release was increased by 50 μM sc1o, whereas IL-23 and IL-8 were not influenced by sc1o during MdDC differentiation (Fig. 1d, Supplemental Fig. 1b). These data indicate that sc1o possibly reduces the potential of MdDC for antigen presentation and phagocytosis.

### Inhibition of dendritic cell activation by sc1o

**Fig. 2:**
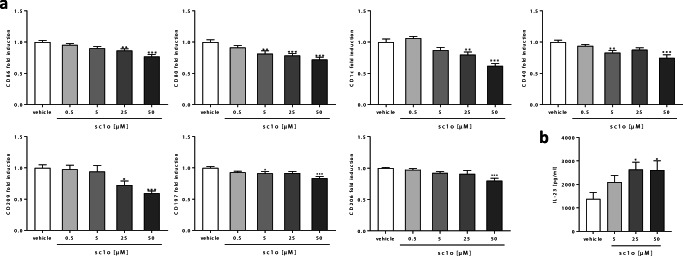
Effect of sc1o on activated MdDCs. Human monocytes were differentiated to MdDCs for 5 days with GM-CSF (10 ng/ml) and IL-4 (10 ng/ml). MdDCs were activated with a mixture of cytokines (TNF-α, IL-6, IL-1β) and PGE2 in the presence or absence of different concentrations of sc1o (0.5–50 μM) or a vehicle (DMSO) for 24 h. (**a**) Surface marker expression was measured with a MACSQuant® Analyser 10 in triplicate. Fold induction of the geometric mean of the fluorescence intensity was calculated by referring treated cells to vehicle controls (*n* = 6 different blood donors in 5 separate experiments). (**b**) Released concentrations of IL-23 in the supernatant were measured with ELISA in triplicate (*n* = 6 different blood donors in 5 separate experiments). For statistical analysis, a one-way ANOVA with Dunnett’s multiple comparisons test (**a**–**b**) was used. Results are presented as means ± standard errors. * *p* < 0.05. ** *p* < 0.01. ***p < 0.001

Furthermore, we investigated to what extent sc1o influences the activation of MdDCs. We found that MdDCs treated with sc1o are characterized by a reduced expression of receptors essential for T cell activation (CD80, CD86, CD40, partially even from 5 μM), antigen presentation (CD1c), phagocytosis (CD206, CD209), and the chemokine receptor CD197 (Fig. 2a). On the other hand, HLA-DR, CD83, CD141, CD54, and CD1a were not noticeably modified (Supplemental Fig. 2a). Twenty-five and 50 μM sc1o induced the release of IL-23, which provokes, among other effects, the differentiation of TH_17_ cells [22] (Fig. 2b). However, several other cyto-/chemokines (IL-12p70, IL-10, IL-6, CXCL8) were not significantly influenced by sc1o treatment (Supplemental Fig. 2b). These data indicate that sc1o possibly impairs antigen presentation, phagocytosis, and the T cell-activating potential of MdDCs.

### Sc1o influenced macrophage differentiation

**Fig. 3:**
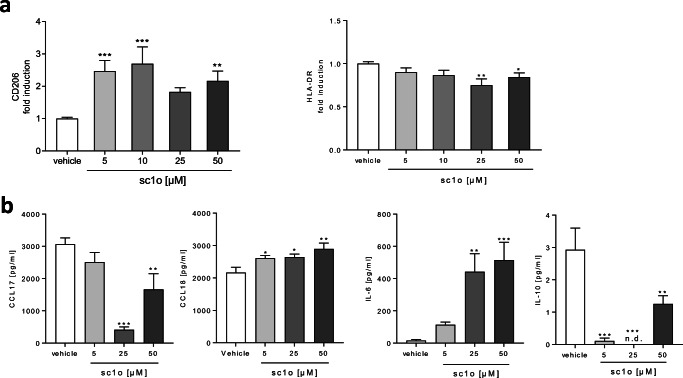
The influence of sc1o on macrophages during differentiation. Human monocytes were isolated and differentiated to MdMs (7 days, 10 ng/ml GM-CSF) in the presence of different concentrations of sc1o (5–50 μM) or vehicle (DMSO). (**a**) Surface marker expression of MdMs was measured with a MACSQuant® Analyser 10 in triplicate. Fold induction of the geometric mean of the fluorescence intensity was calculated by referring treated cells to vehicle controls (*n* = 6–14 different blood donors in 7 separate experiments). (**b**) Released cytokines in the supernatant of MdMs after differentiation with sc1o (5–50 μM) for 7 days. Cytokine concentrations of IL-10/IL-6 were measured with a cytometric bead array and CCL18/CCL17 with ELISA in triplicate (*n* = 4). For statistical analysis, a one-way ANOVA with Dunnett’s multiple comparisons test (**a**–**d**) was used to compare different sc1o concentrations with a vehicle. Results are presented as means ± standard errors. *p < 0.05. **p < 0.01. ***p < 0.001

Besides the process of MdDC differentiation, we examined the influence of sc1o on MdM differentiation. Sc1o significantly increased the expression of the phagocytosis receptor CD206 at the lowest tested concentrations and slightly reduced a receptor relevant to antigen presentation (HLA-DR) (Fig. 3a), whereas sc1o did not modify CD80, CD86, CD163, and TREM2 (Supplemental Fig. 3). Sc1o significantly reduced the release of CCL17 and IL-10. CCL18 and IL-6 were significantly increased (Fig. 3b). These data indicate that, during differentiation, sc1o directs the macrophages towards an anti-inflammatory state, as reflected by the increase in anti-inflammatory marker CD206, and decreases the antigen-presentation marker HLA-DR. However, the environment generated by the sc1o-treated macrophage in releasing cytokines and chemokines is characterized by both anti- and pro-inflammatory influences.

### Sc1o promoted the pro-inflammatory potential of polarized macrophages

**Fig. 4:**
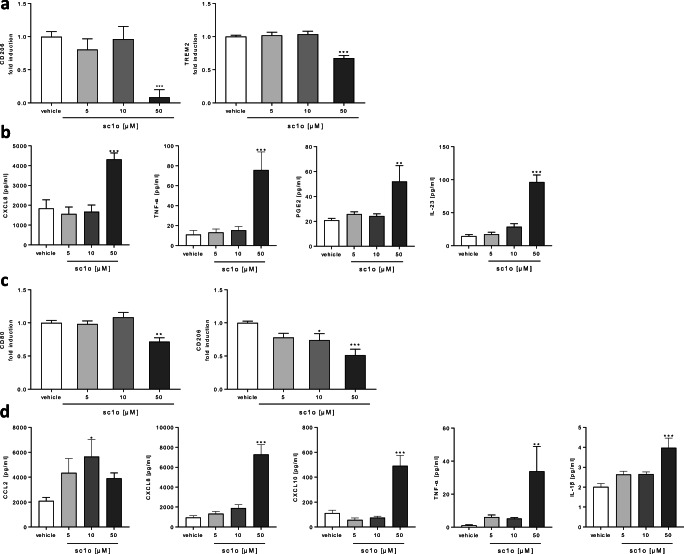
Influence of sc1o on M1- or M2-polarized macrophages. Human monocytes were isolated from buffy coats and differentiated to M1 monocyte-derived macrophages (M1 MdMs) for 7 days using GM-CSF (10 ng/ml) or M2 MdMs using M-CSF (50 ng/ml). After differentiation, cells were polarized with IFN-γ (20 ng/ml) or IL-4 (10 ng/ml) in the presence of different sc1o concentrations (5–50 μM) or a vehicle (DMSO) for 48 h or 24 h to M1 or M2 macrophages, respectively. Surface marker expression and the cytokine/chemokine level of M1 MdMs (**a**, **b**) or M2 MdMs (**c**, **d**) are shown. Surface markers were measured with a MACSQuant® Analyser 10 in triplicate. Fold induction of the geometric mean of the fluorescence intensity was calculated by referring treated cells to vehicle controls (*n* = 6 different blood donors in 3 separate experiments). Released cytokines in the supernatant were determined with either a cytometric bead array or ELISA in triplicate (*n* = 6 different blood donors in 3 separate experiments). For statistical analysis, a one-way ANOVA with Dunnett’s multiple comparisons test (**a**–**d**) was used. Results are presented as means ± standard errors.* *p* < 0.05. ***p < 0.001

Next, we focused on the influence of sc1o on the M1 and M2 polarization of differentiated macrophages. Surface markers regulated during macrophage polarization (e.g. CD80, CD86, CD163, CD206, TREM2, HLA-DR) were determined. In addition, released cytokines and chemokines (e.g. IL-1β, IL-6, IL-10, IL-23, IFN-γ, TNF-α, CCL2, CCL18, CXCL8, CXCL10, PGE_2_) were also detected. Sc1o reduced the expression of the anti-inflammatory markers CD206 and TREM2 in M1 macrophages at 50 μM, whereas CD80, CD86, HLA-DR and CD163 were not modified (Fig. 4a, Supplemental Fig. 4a). Furthermore, 50 μM sc1o significantly upregulated the release of the pro-inflammatory mediators TNF-α, CXCL8, and IL-23, as well as PGE_2_, whereas CCL2, CCL18, CXCL10, IL-10, and IL-1β were not influenced (Fig. 4b, Supplemental Fig. 4a). In the case of M2 macrophages, sc1o significantly reduced the expression of CD80 and CD206 (the latter already at 10 μM), whereas CD86, CD163, TREM2, and HLA-DR were not changed (Fig. 4c, Supplemental Fig. 4b). Moreover, 50 μM sc1o increased the release of the pro-inflammatory TNF-α, CCL2, CXCL8, CXCL10, and IL-1β (Fig. 4d). Sc1o did not influence the release of CCL18, IL-23, IL-10, and PGE_2_ (Supplemental Fig. 4b). Most importantly, these data indicate that sc1o drives polarized M1 and M2 macrophages to a more pro-inflammatory state accompanied by the release of an increased quantity of pro-inflammatory cytokines and chemokines, thereby generating a pro-inflammatory environment.

### Sc1o influenced energy metabolism of myeloid cells

**Fig. 5:**
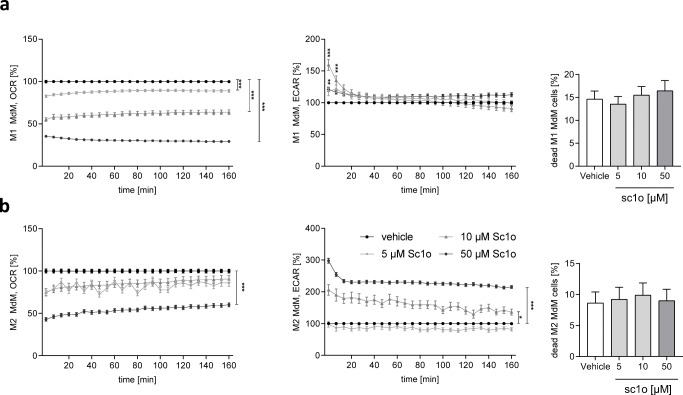
Sc1o influences the energy metabolism of polarized MdMs. The influence of sc1o on the energy metabolism and cell death on polarized M1 MdMs (**a**) and polarized M2 MdMs (**b**). Human monocytes were isolated from buffy coats, stimulated (10 ng/ml GM-CSF (M1) or 50 ng/ml M-CSF (M2)), and incubated for 7 days. After differentiation, MdMs were further polarized to M1 and M2 MdMs using IFN-γ (20 ng/ml) or IL-4 (10 ng/ml) in the presence of 5, 10, or 50 μM sc1o for 48 h or 24 h, respectively. Cell death was determined with the Zombie Aqua™ Fixable Viability Kit and analysed by flow cytometry. The extracellular acidification rate (ECAR) and oxygen consumption rate (OCR) were measured with the Seahorse XFe96 analyser (Agilent, Waldbronn, Germany) over a total time period of 160 min. Sc1o-treated cells were compared to vehicle- (DMSO) treated control. To compare ECAR and OCR of sc1o-treated cells to vehicle treated control, a two-way ANOVA with Bonferroni’s multiple comparisons test was used. Results are presented as means ± standard errors. *p < 0.05. ***p < 0.001

The predominant energy source of M1 macrophages is glycolysis, whereas M2 macrophages use oxidative phosphorylation to meet their energy requirements [23]. Since sc1o increased the viability of MdMs and drives macrophages towards a more pro-inflammatory condition, we investigated whether this was due to modified energy metabolism. For this, OCR, a marker for mitochondrial respiration, and ECAR, a marker for glycolysis, were determined (Fig. 5). In M1-polarized macrophages, sc1o transiently reduced OCR depending on concentration (Fig. 5a), while ECAR was not notably modified. In M2-polarized macrophages, 50 μM sc1o reduced OCR, while ECAR was significantly increased by 10 and 50 μM sc1o (Fig. 5b). Since increasing metabolic rates can be linked to cell death [24], we investigated whether sc1o induces cell death in M1 and M2 MdM cells. However, sc1o reveal no cytotoxic effects in M1 and M2 MdM cells (Fig. 5a, b, extreme right). These data indicate that sc1o increased glycolysis in M2 macrophages, whereas oxidative phosphorylation was suppressed in M1 and M2 macrophages.

## Discussion

**Fig. 6:**
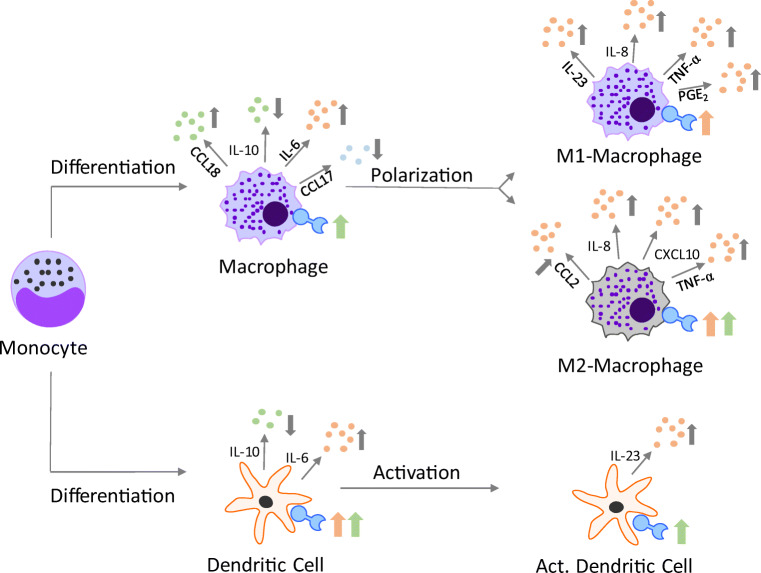
Overview of sc1o (50 μM)-mediated effects on myeloid cells. The effects of sc1o (50 μM) during the differentiation of monocytes to macrophages (MdM) and dendritic cells (MdDC), during the polarization of macrophages to M1 or M2 MdM, and during the activation of monocyte-derived dendritic cells (MdDC) are shown. Only effects with an alteration of at least 20% are shown. Predominant pro-inflammatory cytokines/chemokines are labelled in bright red; predominant anti-inflammatory cytokines/chemokines are shown in bright green; and cytokines/chemokines with pro- and anti-inflammatory potential are shown in bright blue. The directions of the arrows indicate a decrease (↓) or increase (↑). For a better overview and since the regulation of the surface marker was not as strong as the cytokine/chemokine alteration, the changes in the surface marker are shown as tendencies. Therefore, if the majority of the surface markers point to pro-inflammatory regulation, a pink arrow is shown; if the majority point to an anti-inflammatory regulation, a green arrow is shown; and if they were equal, both arrows are shown

Initially, sc1o was identified as a new and promising candidate for the treatment of malaria [20]. Beside its direct anti-parasitic effects, we surmised that sc1o might have an additional immune modulating character. In the current study, we could show that sc1o up to 300 μM did not reduce viability in MdDCs and MdMs. Moreover, 50 μM sc1o amplified the inflammatory potential of M1 MdMs, directing M2 MdMs towards an M1 phenotype and suppressing the activation of MdDCs, particularly their expression of CD40 and CD80. The monitored effects are summarized in Fig. 6.

We propose that these effects of sc1o are linked to metabolic alterations induced by sc1o. Sc1o amplifies the M1 phenotype of M1 MdMs and shifts M2 MdMs to the M1 phenotype. The metabolic pathways in M1 and M2 macrophages are differentially activated [25]. M1 macrophages are characterized by upregulated glycolysis, impairment of oxidative phosphorylation, and disruption of the Krebs cycle at two steps, after formation of citrate and succinate [26, 27]. Citrate is used for fatty acid biosynthesis, which allows for the increased synthesis of inflammatory prostaglandins, and for succinate, which activates the transcription factor HIF-1α, a regulator of a wide range of genes, including IL-1β, CCL2, and CXCL8 [26, 28, 29]. Therefore, one would expect that sc1o, in shifting M2 MdMs to an M1 phenotype, should be characterized by an increased release of IL-1β, CXCL8, and CCL2, by increased glycolysis, and a reduction of oxidative phosphorylation measured. Indeed, we observed such an increased expression of IL-1β, CXCL8, and CCL2, an increase in glycolysis (ECAR), and an impairment of oxidative phosphorylation (OCR) in sc1o-treated M2 MdMs. The observed increase in PGE_2_ levels in sc1o-treated M1 MdMs, in which sc1o amplifies the M1 phenotype, might indicate that the Krebs cycle is disrupted and citrate is used to synthesize PGE_2_. Additionally, the OCR, which is a metabolic pathway for M2 MdMs, was significantly decreased in M1 MdMs, while ECAR was not affected, probably due to the pre-existing high level of ECAR. These data indicate that sc1o probably alters the energy metabolism of MdMs, leading to the promotion of an M1-phenotype in M1 and M2 MdMs.

In malaria infections, an imbalance between the M1 and M2 phenotype in monocytes in favour of the M2 phenotype is observed [7]. The strengthening of the M2 phenotype is probably due to haemozoin, a malaria pigment that is involved in the breakdown of human haemoglobin by the parasites and that is linked to numerous immunological effects [8]. The administration of haemozoin increased the expression of the resolution-promoting surface receptor CD206 as well as the secretion of the cyto- and chemokines IL-10, CCL17, and CCL1 in human monocytes [7]. Furthermore, the M1 phenotype is suppressed due to less NO and ROS formation. The involvement of the signalling pathways, p38-mitogen-activated protein kinase (p38 MAPK), phosphoinositide-3-kinase (PI3K)/protein kinase B, and NFκB in the enhancement of the M2 phenotype was demonstrated. The M2 phenotype is further characterized by the expression of the surface marker CD163 and the formation of the enzyme arginase 1. Investigations in children infected with malaria showed increased levels of arginase 1 and IL-10, reduced amounts of NO, and increased expression of M2 markers (CD163, CD206) [30]. The malaria drugs chloroquine and artemisinin reduced the haemozoin-induced M2 phenotype by reducing IL-10 and CD206 [7]. By strengthening the M1 phenotype and weakening the M2 phenotype of macrophages by steroid substance 1o, this could counteract the effects of haemozoin and improve the course of malaria, similar to chloroquine and artemisinin. However, it should be remembered that increased inflammation in malaria could also lead to negative effects such as cerebral malaria. This is usually treated with anti-inflammatory drugs such as statins [31]. A strengthening of the pro-inflammatory aspects by sc1o could therefore counteract this and prohibit the administration of sc1o. Further studies on the immune modulatory effects of sc1o in the presence of *P. falciparum* are needed to clarify these issues.

The increased release of TNF-α, IL-23, CCL2, CXCL8, and CXCL10 by sc1o-treated MdMs may contribute to a pro-inflammatory environment at the infection site and thereby promote the immune response against pathogens via several mechanisms. With an amplified release of IL-23, sc1o could induce the formation of TH_17_ cells, which play a crucial role in host defence against a variety of pathogens, including bacteria and viruses [32]. The presence of TH_17_ cells was mainly observed in the liver during the acute erythrocytic stage of *Plasmodium chabaudi* infection. However, IL-17A-deficient mice showed no significant alterations in the course of *Plasmodium chabaudi* infection. Therefore, despite activation, TH_17_ cells have so far not been demonstrated to exert a defined role during *Plasmodium* infections [33]. Consequently, as sc1o also tended to decrease dendritic cell potential for antigen presentation to T cells, this would not be of much relevance in a malaria-infected host, except for possible effects on T cell activation with long-term treatment.

The immune modulatory effects of sc1o were observed at concentrations of 25 or 50 μM. However, previous studies showed a low permeability of sc1o in the Caco-2 cell barrier assay indicating that only a limited amount of sc1o passes through the cell barrier, and possibly also only a small amount of sc1o is absorbed by the cells [21]. In vivo studies revealed a plasma level of 2 μM after i.p. dosing at 100 mg/kg [20]. One possibility to increase the bioavailability of sc1o is to use a suitable formulation. Interestingly, pharmacokinetic studies with formulated sc1o revealed an increase in plasma levels in comparison to sc1o without any carrier excipients (unpublished data). Unfortunately, the formulation only increases the concentration of compounds in vivo not in in vitro cell culture. Thus, further studies are needed to investigate whether the formulated sc1o shows immune modulatory potency in vivo.

Taken together, the modulation of MdMs towards a pro-inflammatory phenotype by sc1o might be an efficient way to counteract the capacity of *P. falciparum* to escape immune clearance mechanisms. Promoting host defence mechanisms might be particularly efficient in the early stages of the disease to minimize the later risk of enhanced cerebral malaria due to inflammatory processes. However, the sc1o concentrations needed for anti-parasitic effects are lower than those for the immune modulatory effects. Therefore, if the immune modulatory potential of sc1o is likely to be therapeutically useful, as in counteracting the immunosuppressing effects of *P. falciparum* or in parasite resistance, the sc1o dosages will need adjusting.

## Materials and methods

### Cells and reagents

Primary human monocytes, macrophages, and dendritic cells were cultured in RPMI1640 GlutaMAX medium supplemented with 10% FCS. All media contain 1% penicillin/streptomycin, and the cells were cultured at 37 °C in a 5% CO_2_ atmosphere. Sc1o was dissolved in DMSO and further diluted in media (*c*_stock_ = 25 mM, maximal DMSO concentration during experiments 0.3% v/v EDTA was from Sigma Aldrich (Schnelldorf, Germany). Human FcR blocking reagent, bovine serum albumin, human CD14 MicroBeads, human GM-CSF, human M-CSF, IL-4, and all antibodies for surface staining except CD197 from BioLegend (Fell, Germany) were from Miltenyi Biotec (Bergisch Gladbach, Germany). Human IL-6, IFN-γ, IL-1β, and TNF-α were from PeproTech (Hamburg, Germany). PGE_2_ and Accutase solution were from Merck (Darmstadt, Germany).

### Cell viability assay

To determine the cell viability of human MdMs and MdDCs, the Orangu™ assay was used. After isolation of CD14^+^ cells, MdMs and MdDCs were differentiated as described in the following sections. After differentiation, 1 × 10^5^ cells were seeded in triplicate in 96-well plates. Different concentrations of sc1o (0.5–300 μM) or vehicle (DMSO) were added, and cells were incubated at 37 °C and 5% CO_2_. After 48 h of incubation at 37 °C and 5% CO_2_, 10 μl of Orangu™ cell counting solution (Cell Guidance Systems, Cambridge, UK) was added and incubated for 120 min at 37 °C and 5% CO_2_. After incubation, absorbance was measured at a wavelength of 450 nm with a reference at 650 nm at an EnSpire® 2300 Multimode Plate Reader (Perkin Elmer, Lübeck, Germany). To calculate cell viability in the Orangu™ assay, the absorbance of vehicle-treated cells was set to 100%, and the sc1o-treated samples were correlated to them.

### Isolation of human CD14^+^ cells

Human peripheral blood mononuclear cells were isolated from buffy coats using density gradient. For this, 25 ml of blood from healthy donors (German Red Cross, Frankfurt, Germany) were mixed with the same amount of Hank’s balanced salt solution (Thermo Fisher Scientific, Oberhausen, Germany) and were layered over 15 ml of Biocoll (Merck, Darmstadt, Germany) in Sep-Mate™-50 tubes (Stemcell Technologies, Cologne, Germany). Tubes were centrifuged (1200 *g*, 10 min, RT), and human peripheral blood mononuclear cells were isolated from the interphase, washed with 2 mM EDTA/PBS four times and counted using a MACSQuant® Analyser 10 flow cytometer (Miltenyi Biotec, Bergisch Gladbach, Germany). Defined amounts of cells were dissolved in 0.5% bovine serum albumin/2 mM EDTA/PBS and incubated with 25% (v/v) human CD14 MicroBeads for 15 min at 4 °C. Afterwards magnetically labelled cells were separated via magnetic cell separation with LS columns (Miltenyi Biotec, Bergisch Gladbach, Germany) according to the manufacturer’s protocol. Cell count was determined with flow cytometry.

### Differentiation of human macrophages and dendritic cells

For differentiation of human MdM or MdDC, 0.5 × 10^6^ or 0.9 × 10^6^ CD14^+^ cells/well, respectively, were cultivated in 48-well plates in the presence of different concentrations of sc1o (0.5–50 μM) or vehicle (DMSO) in triplicate. After 30 min of pre-incubation for the differentiation of MdM, 10 ng/ml of human GM-CSF was added, and for MdDC, 10 ng/ml human GM-CSF/10 ng/ml human IL-4 were added. MdMs were differentiated for 7 days, while MdDCs were incubated for 5 days. Medium including sc1o and growth factors was completely changed for MdM after 3 days. After incubation, cells were centrifuged (300 *g*, 5 min, RT), and supernatant was stored at − 80 °C for ELISA and a cytometric bead array. Cells were washed with PBS and harvested by using Accutase (15 min, 37 °C, 5% CO_2_). Cell count was determined with flow cytometry.

### Polarization of human macrophages

For polarization, 7.5 × 10^6^ human CD14^+^ cells were seeded in T-75 flasks (Thermo Fisher Scientific, Oberhausen, Germany). For subsequent M1 polarization, 10 ng/ml of human GM-CSF was added, while 50 ng/ml of human M-CSF was used for subsequent M2 polarization. During this first differentiation, no sc1o was added to the cells to provide an unmodified process. After 7 days, cells were washed and incubated with 5 ml of Accutase (15 min, 37 °C, 5% CO_2_). Afterwards 15 ml medium was added, and cells were scraped off. Cell count was determined using flow cytometry. For polarization, 5 × 10^5^ cells were seeded in triplicate in 48-well plates, and sc1o (5–50 μM) or vehicle (DMSO) was added. After 30 min of pre-incubation (37 °C, 5% CO_2_), 20 ng/ml of human IFN-γ (M1) or 10 ng/ml of human IL-4 (M2) was added for polarization, respectively. Cells were harvested after 24 h (M2) or 48 h (M1) of incubation (37 °C, 5% CO_2_). The quantity of dead cells was calculated using the Zombie Aqua™ Fixable Viability Kit (BioLegend, Fell, Germany).

### Activation of human dendritic cells

To study the activation of dendritic cells, other maturing conditions were selected than for the study of their differentiation. For differentiating monocytes to dendritic cells, 1.5 × 10^7^ isolated CD14^+^ cells were seeded in T-75 flasks with 50 ng/ml of human GM-CSF and 50 ng/ml of human IL-4. After 5 days of differentiation without sc1o, cells were harvested and seeded in triplicate, with 0.9 × 10^6^ cells/well in 48-well plates. Sc1o (0.5–50 μM) or vehicle (DMSO) was added, and after 30 min of pre-incubation (37 °C, 5% CO_2_), 5 ng/ml human TNF-α, 5 ng/ml human IL-6, 5 ng/ml human IL-1β, and 500 ng/ml PGE2 were added. Cells were incubated for 24 h (37 °C, 5% CO_2_) and harvested for analysis via flow cytometry.

### Flow cytometry

For flow cytometry, 1.5–2 × 10^5^ cells of each sample were analysed. All steps were performed on ice if not stated otherwise. First, non-specific antibody binding to Fc-γ receptors was blocked with human FcR blocking reagent for 15 min at 4 °C. For discriminating living and dead cells, the Zombie Aqua™ fixable viability kit (1:500 dilution, BioLegend, San Diego, CA, USA) was used according to the manufacturer’s protocol. After viability, samples were stained with a cocktail of different surface marker antibodies (for MdM, CD14, CD80, CD86, CD163, CD206, TREM2, HLA-DR and for MdDC, CD11c, CD54, CD1a, CD1c, HLA-DR, CD40, CD83, CD141, CD197, CD206, CD209, CD80, CD86) for 15 min at 4 °C. Afterwards, 250 μl of 10% FCS/PBS was added, and cells were centrifuged (300 *g*, 5 min, 4 °C). Cells were suspended in 100 μl PBS and measured with a MACSQuant® Analyser 10 flow cytometer. The geometric mean of the fluorescence intensity was calculated using FlowJo software v10 (Treestar, Ashland, TN, USA). Fold induction was calculated by referring treated cells to DMSO controls. Exemplary gating strategy was displayed in Supplement Fig. 5.

### Determination of chemo- and cytokines in the supernatant

For detecting chemo- and cytokines in the supernatant of differentiated, polarized, or activated MdM and MdDC, the cytometric bead array (BD Bioscience, Heidelberg, Germany) was used for TNF-α, IL-1β, IL-6, IL-10, CCL2, CXCL8, and CXCL10, while ELISAs were used to detect IL-23 (Thermo Fisher, Waltham, MA, USA), CCL17 (BioLegend, Fell, Germany), CCL18 (Boster Biological Technology, Pleasanton, CA, USA), and PGE2 (Enzo, Lörrach, Germany). Cytometric bead array was performed according to the manufacturer’s protocol. In a deviation from protocol, only half of the capture and detection beads and 25 μl of the supernatant were used. ELISAs were performed strictly according to the manufacturer’s protocol. For the ELISAs, 100 μl of the supernatant was used.

### Determination of cell energy metabolism

To analyse the extracellular acidification rate (ECAR) and oxygen consumption rate (OCR) of polarized macrophages, the Seahorse XFe96 FluxPak (Agilent, Waldbronn, Germany) was used as recommended by the manufacturer. CD14^+^ cells were isolated, and macrophages were polarized as previously described. Macrophages were polarized in the presence of 5, 10, or 50 μM sc1o. After polarization, cells were washed with Seahorse XF RPMI medium pH 7.4 (Agilent, Waldbronn) and incubated for 60 min at 37 °C. OCR and ECAR were measured for a total period of 160 min in the absence of sc1o. Cells were measured as octuplicates with 3 × 10^4^ cells per well with the Seahorse XFe96 Analyser (Agilent, Waldbronn, Germany) and analysed with Wave Software (Agilent, Waldbronn, Germany). Values of vehicle (DMSO)-treated cells were set to 100% and sc1o-treated cells referred to this.

### Statistics

Results are presented as means ± standard errors. The data was analysed with one-way or two-way ANOVA and with Dunnett’s comparison test. For all calculations and creation of graphs, GraphPad Prism 8 was used, and *p* < 0.05 was considered the threshold for significance.

## Supplementary information

ESM 1 (PDF 248 kb)

ESM 2 (PDF 328 kb)

ESM 3 (PDF 240 kb)

ESM 4 (PDF 350 kb)

ESM 5 (PDF 235 kb)

## Data Availability

Data are available in request to corresponding author.

## Abbreviations

ANOVA: Analysis of variance
ECAR: Extracellular acidification rate
FCS: Foetal calf serum
GM-CSF: Granulocyte macrophage colony-stimulating factor
IFN: Interferon
IL: Interleukin
MdM: Monocyte-derived macrophage
MdDC: Monocyte-derived dendritic cell
OCR: Oxygen consumption rate
PBS: Phosphate-buffered saline
sc1o: Steroid compound 1o
