# Supplementary material for: The immunomodulatory potential of the arylmethylaminosteroid sc1o
Source: J Mol Med (Berl). 2020 Dec 17;99(2):261–72. doi: 10.1007/s00109-020-02024-4 (PMC7819914; doi:10.1007/s00109-020-02024-4)
Supplement: Supplementary file 1 — (PDF 248 kb) [file 109_2020_2024_MOESM1_ESM.pdf]

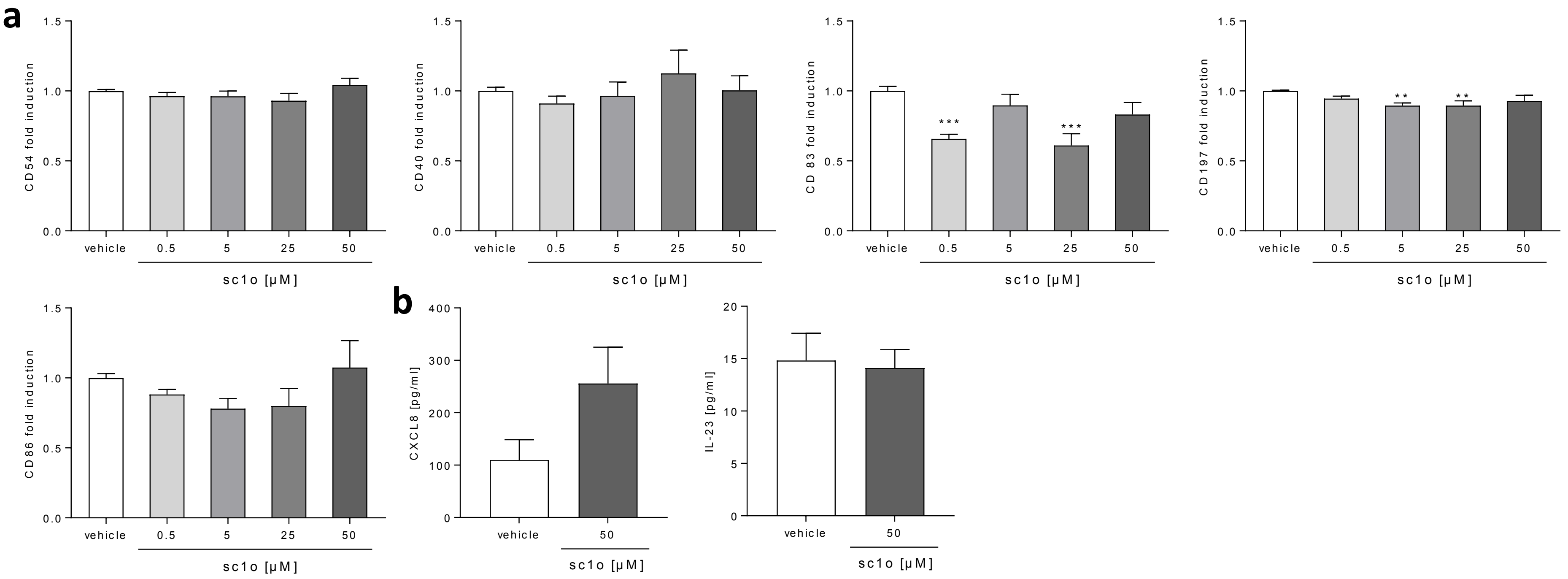

Supplemental Figure 1: Effect of steroid compound 1o (sc1o) on surface markers and cytokine/chemokine release of monocyte-derived dendritic cells (MdDCs). Human monocytes were differentiated to MdDCs for 5 days with GM-CSF (10 ng/ml) and IL-4 (10 ng/ml) in the presence or absence of different concentrations of sc1o (0.5, 5, 25, 50 µM) or a vehicle (DMSO). a) Surface marker expression was measured with a MACSQuant® Analyser 10 in triplicate. Fold induction of the geometric mean of the fluorescence intensity was calculated by referring treated cells to vehicle controls (n = 6-11). b) Released concentrations of IL-23 and CXCL-8 in the supernatant were measured with a cytometric bead array or ELISA in triplicate (n = 4). For statistical analysis, a one-way ANOVA with Dunnett's multiple comparisons test (a) or unpaired t-test (b) was used. **p<0.01. ***p<0.001
