# Supplementary material for: The immunomodulatory potential of the arylmethylaminosteroid sc1o
Source: J Mol Med (Berl). 2020 Dec 17;99(2):261–72. doi: 10.1007/s00109-020-02024-4 (PMC7819914; doi:10.1007/s00109-020-02024-4)
Supplement: Supplementary file 2 — (PDF 328 kb) [file 109_2020_2024_MOESM2_ESM.pdf]

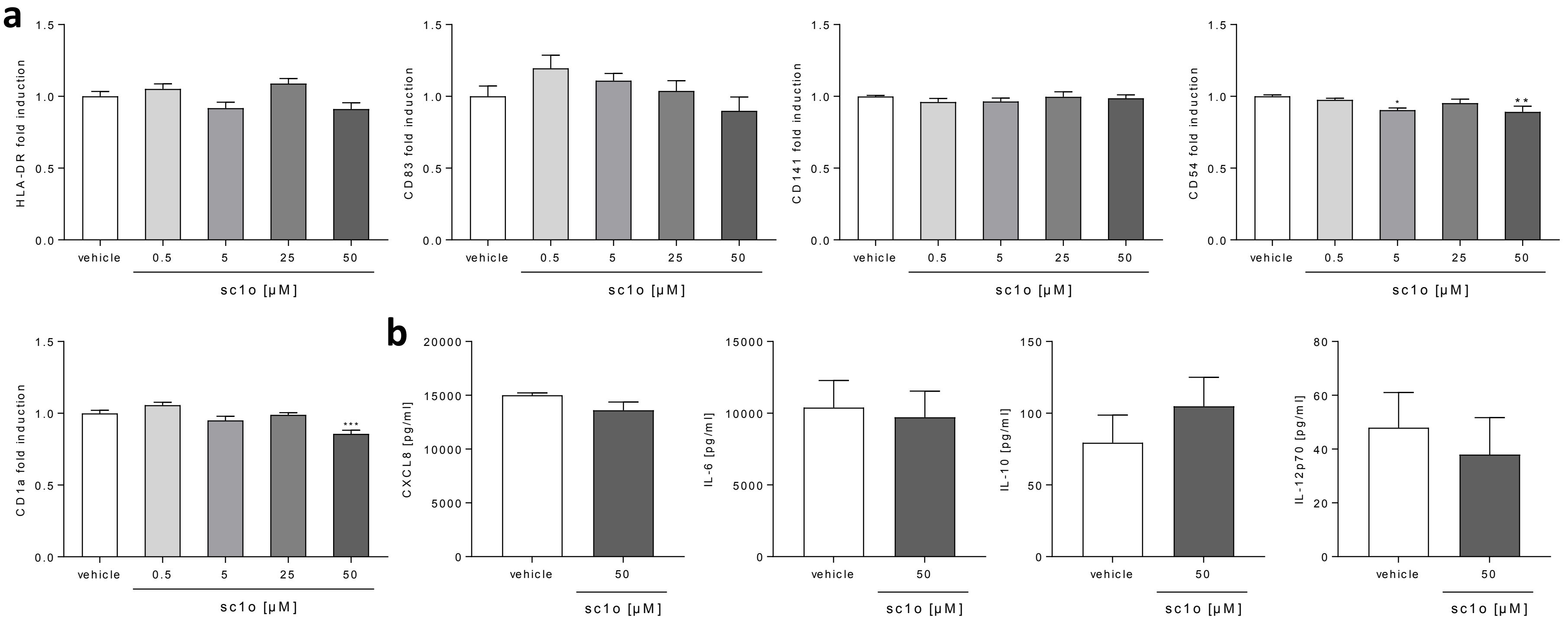

Supplemental Figure 2: Effect of steroid compound 1o (sc1o) on surface markers and cytokine/chemokine release of activated monocyte-derived dendritic cells (aMdDCs). Human monocytes were differentiated to MdDCs for 5 days with GM-CSF (c = 10 ng/ml) and IL-4 (c = 10 ng/ml). Afterwards MdDCs were activated with a mix of cytokines (TNF-α, IL-6, IL-1β, PGE_2) in the presence or absence of different concentrations of sc1o (0.5, 5, 25, 50 µM) or a vehide (DMSO) for 24 h. a) Surface marker expression was measured with a MACSQuant® Analyser 10 in triplicate. Fold induction of the geometric mean of the fluorescence intensity was calculated by referring treated cells to vehicle controls (n = 6). b) Released concentrations of IL-12p70, IL-10, IL-8, and IL-6 in the supernatant were measured with a cytometric bead array in triplicate (n = 6). For statistical analysis, a one-way ANOVA with Dunnett's multiple comparisons test (a) or an unpaired *t*-test (b) was used. * $p<0.05$. ** $p<0.01$. *** $p<0.001$.
