# Supplementary material for: The immunomodulatory potential of the arylmethylaminosteroid sc1o
Source: J Mol Med (Berl). 2020 Dec 17;99(2):261–72. doi: 10.1007/s00109-020-02024-4 (PMC7819914; doi:10.1007/s00109-020-02024-4)
Supplement: Supplementary file 3 — (PDF 240 kb) [file 109_2020_2024_MOESM3_ESM.pdf]

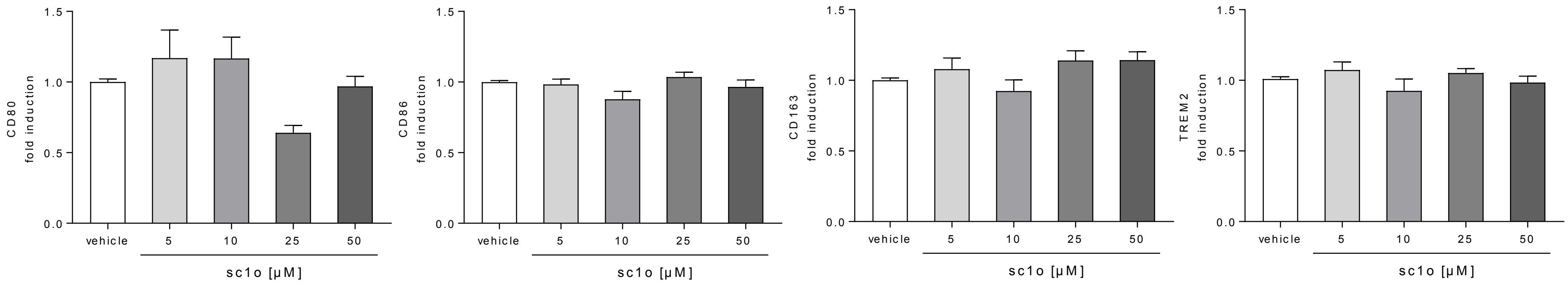

Supplemental Figure 3: Effect of steroid compound 1o (sc1o) on surface markers of monocyte-derived macrophages (MdMs). Human monocytes were isolated from buffy coats and stimulated with GM-CSF (10 ng/ml) in the presence or absence of different concentrations of sc1o or a vehicle (DMSO) as indicated for 7 days. MdM medium was completely renewed after 3 days. Surface marker expression of MdMs was measured with a MACSQuant® Analyser 10 in triplicate. Fold induction of the geometric mean of the fluorescence intensity was calculated by referring treated cells to vehicle controls (n = 6-14). For statistical analysis, a one-way ANOVA with Dunnett’s multiple comparisons test was used.
