# Supplementary figures and images for: The immunomodulatory potential of the arylmethylaminosteroid sc1o
Source: J Mol Med (Berl). 2020 Dec 17;99(2):261–72. doi: 10.1007/s00109-020-02024-4 (PMC7819914; doi:10.1007/s00109-020-02024-4)

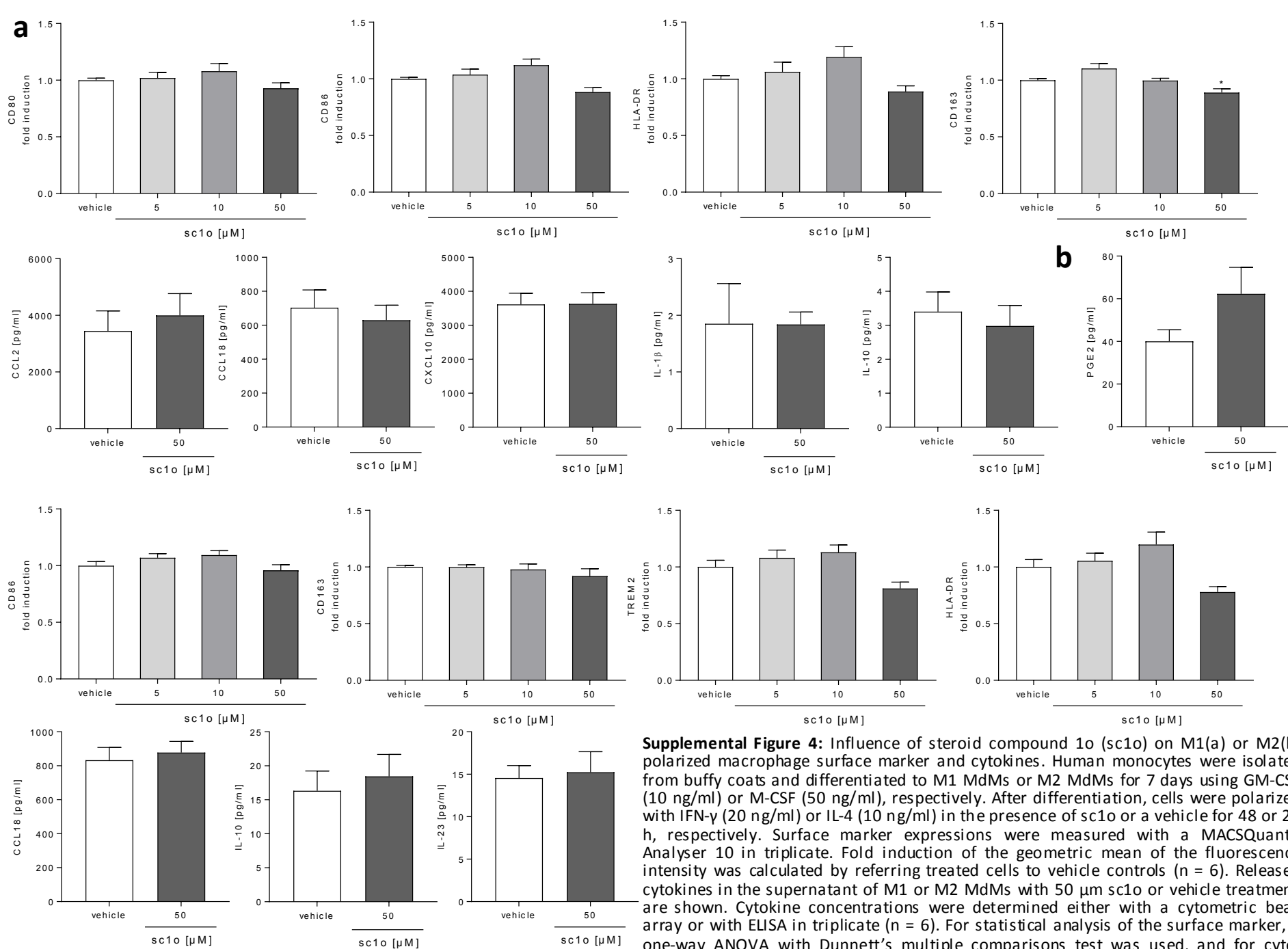

Supplement: Supplementary file 4 — (PDF 350 kb) [file 109_2020_2024_MOESM4_ESM.pdf]
