# Supplementary material for: The immunomodulatory potential of the arylmethylaminosteroid sc1o
Source: J Mol Med (Berl). 2020 Dec 17;99(2):261–72. doi: 10.1007/s00109-020-02024-4 (PMC7819914; doi:10.1007/s00109-020-02024-4)
Supplement: Supplementary file 5 — (PDF 235 kb) [file 109_2020_2024_MOESM5_ESM.pdf]

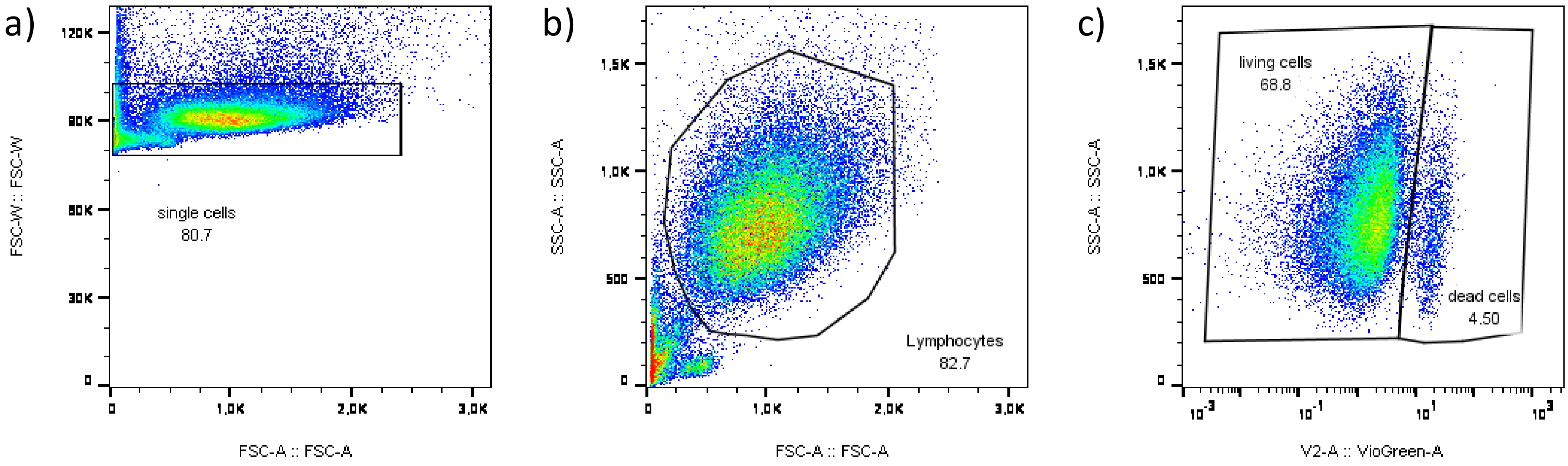

Supplemental Figure 5: Gating strategy for data presented in Fig. 1-4. a) In the first step, only single cells were used for the next analysis step using FSC-A/FSC-W. b) FSC-A/SSC-A was used to separate cells of interest from cell debris. c) Zombie Aqua™ fixable viability kit was used to stain dead cells (V2-A: VioGreen positive). Position of the gates was determined on the basis of negative controls. Only living cells (V2-A: VioGreen negative) were used for the analysis of the geometric mean of expressed surface markers.
